# The structure of *Mycobacteria *2*C*-methyl-D-erythritol-2,4-cyclodiphosphate synthase, an essential enzyme, provides a platform for drug discovery

**DOI:** 10.1186/1472-6807-7-68

**Published:** 2007-10-23

**Authors:** Lori Buetow, Amanda C Brown, Tanya Parish, William N Hunter

**Affiliations:** 1 Division of Biological Chemistry and Molecular Microbiology, College of Life Sciences, University of Dundee, Dundee DD1 5EH, UK; 2 Centre for Infectious Disease, Institute for Cell and Molecular Science, Barts and the London, 4 Newark Street, London E1 2AT, UK

## Abstract

**Background:**

The prevalence of tuberculosis, the prolonged and expensive treatment that this disease requires and an increase in drug resistance indicate an urgent need for new treatments. The 1-deoxy-D-xylulose 5-phosphate pathway of isoprenoid precursor biosynthesis is an attractive chemotherapeutic target because it occurs in many pathogens, including *Mycobacterium tuberculosis*, and is absent from humans. To underpin future drug development it is important to assess which enzymes in this biosynthetic pathway are essential in the actual pathogens and to characterize them.

**Results:**

The fifth enzyme of this pathway, encoded by *ispF*, is 2*C*-methyl-D-erythritol-2,4-cyclodiphosphate synthase (IspF). A two-step recombination strategy was used to construct *ispF *deletion mutants in *M. tuberculosis *but only wild-type double crossover strains were isolated. The chromosomal copy could be deleted when a second functional copy was provided on an integrating plasmid, demonstrating that *ispF *is an essential gene under the conditions tested thereby confirming its potential as a drug target. We attempted structure determination of the *M. tuberculosis *enzyme (*Mt*IspF), but failed to obtain crystals. We instead analyzed the orthologue *M. smegmatis *IspF (*Ms*IspF), sharing 73% amino acid sequence identity, at 2.2 Å resolution. The high level of sequence conservation is particularly pronounced in and around the active site. *Ms*IspF is a trimer with a hydrophobic cavity at its center that contains density consistent with diphosphate-containing isoprenoids. The active site, created by two subunits, comprises a rigid CDP-Zn^2+ ^binding pocket with a flexible loop to position the 2*C*-methyl-D-erythritol moiety of substrate. Sequence-structure comparisons indicate that the active site and interactions with ligands are highly conserved.

**Conclusion:**

Our study genetically validates *Mt*IspF as a therapeutic target and provides a model system for structure-based ligand design.

## Background

Approximately one-third of the world's population is infected with *Mycobacterium tuberculosis*, the causative agent of tuberculosis and 2005, almost 9 million cases of tuberculosis emerged, resulting in an estimated 1.6 million deaths [1]. Typical treatments require combination drug therapies taken over a period of 6–9 months. The global economic burden of tuberculosis amounts to approximately $12 billion annually. The need for novel chemotherapeutics in the treatment of infection by *M. tuberculosis *is clearly demonstrated by its high infectivity rate and prolonged and extensive therapy requirements.

The isoprenoid biosynthesis pathways are attractive, established targets for chemotherapeutic treatment [2,3]. Isoprenoids are building blocks for several biologically or commercially important compounds, including steroids, flavoring compounds like limonene, and natural medicinal products like taxol [4]. Cells are dependent on isoprenoid derivatives for critical functions like growth, hormone-based signaling, differentiation, maintenance of homeostasis, and electron transport in respiration and photosynthesis [4]. In *Mycobacteria *species, isoprenoid biosynthesis is particularly important for the synthesis of the cell wall, including mycolic acids and lipoarabinomannan [5]. The universal precursors of isoprenoids are the isomers isopentenyl pyrophosphate (IPP) and dimethylallyl pyrophosphate (DMAPP). Synthesis of these precursors occurs via two distinct biochemical pathways. In mammals, fungi, the cytoplasm of plants, and archaebacteria, synthesis occurs via the mevalonate pathway [6], and, in chloroplasts, algae, cyanobacteria, apicomplexa and most eubacteria (including *M. tuberculosis*), via the 1-deoxy-D-xylulose 5-phosphate (DOXP) or non-mevalonate pathway [7-11]. Fosmidomycin is an inhibitor of the third enzyme in the DOXP pathway, 1-deoxy-D-xylulose 5-phosphate reductoisomerase, and has been used against infections by *Plasmodium *species [3,12]. Since the compound is a clinically approved antibacterial agent then there is chemical validation of this stage of the pathway for drug development. Recently, the crystal structure of the *M. tuberculosis *reductoisomerase has been determined opening up routes to structure-based inhibitor discovery methods targeting that particular stage of the pathway [13].

**Figure 1:**
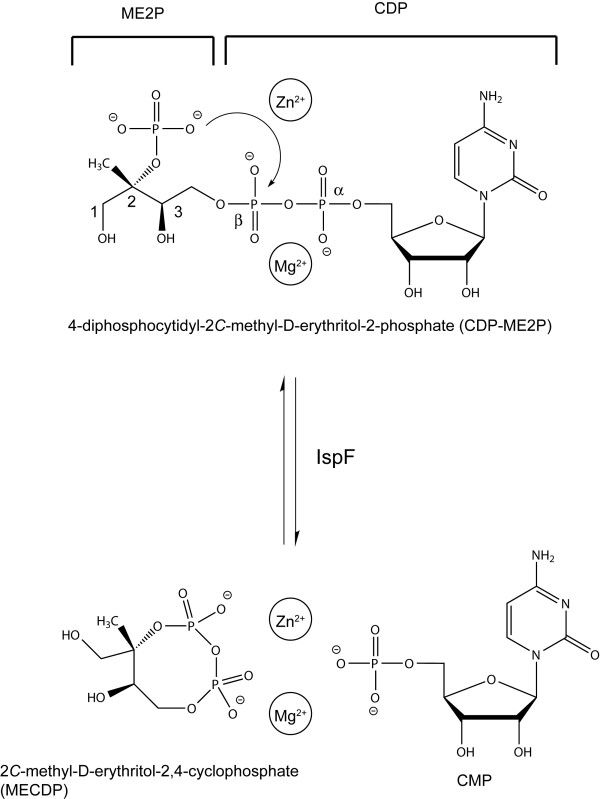
The IspF reaction. IspF catalyzes the formation of 2*C*-methyl-D-erythritol 2,4-cyclodiphosphate (MECDP) and CMP by an internal direct attack of the 2-phosphate group on the β-phosphate of the substrate, 4-diphosphocytidyl-2*C*-methyl-D-erythritol-2-phosphate (CDP-ME2P). The reaction is dependent on divalent cations (Zn^2+ ^and under physiological conditions Mg^2+^).

Eight enzymes are involved in the synthesis of IPP and DMAPP via the DOXP pathway [8,14]. IspF, or 2*C*-methyl-D-erythritol-2,4-cylodiphosphate (MECDP) synthase, is the fifth enzyme of the pathway. Structural and biochemical studies, in particular on the *Escherichia coli *enzyme (*Ec*IspF), demonstrate that IspF directs an intramolecular attack of the 2-phosphate on the internal β-phosphate of the substrate, 4-diphosphocytidyl-2*C*-methyl-D-erythritol-2-phosphate (CDP-ME2P), to form MECDP and CMP (Figure 1). IspF depends on two divalent cations to orient and polarize the substrate during catalysis [15-18] In Gram-negative bacteria and *Mycobacteria *species, *ispF *is found in a putative operon with *ispD*, which encodes the third enzyme in the DOXP pathway [14]. Genetic studies indicate that *ispF *is essential in *E. coli *as well as *Bacillus subtilis *[8,19,20] and partial depletion of *ispF *in these bacteria increases sensitivity to cell wall-active antibiotics [19]. In larger genomic scale hybridization studies, failure to insert a transposon into the *ispF *gene also suggests it is essential in *Haemophilus influenzae *[21] and *M. tuberculosis *[22].

These observations, in conjunction with the absence of this enzyme from humans, demonstrate the importance of IspF as a novel target for drug discovery. The structure of *Ec*IspF has provided a model for rational ligand design [23] and a high throughput screen has been developed to enable ligand discovery [24]. Little information is available for *M. tuberculosis *IspF (*Mt*IspF) and earlier genetic studies only suggest that *ispF *is essential in this organism. Here, we prove that *ispF *is essential in *M. tuberculosis*. Furthermore, for use as a model in structure-based ligand design, we present a structure of the orthologue, *Mycobacterium smegmatis *IspF (*Ms*IspF), bound to CDP.

## Results and discussion

### IspF essentiality in *M. tuberculosis*

**Figure 2:**
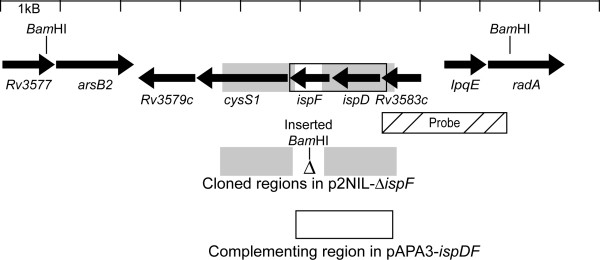
*M. tuberculosis ispF *is essential. Map of the *ispF *genomic region in the wild type and the deletion allele. Regions amplified for the delivery and complementing vectors, restriction sites (intragenic and introduced) and probe location are indicated.

**Figure 3:**
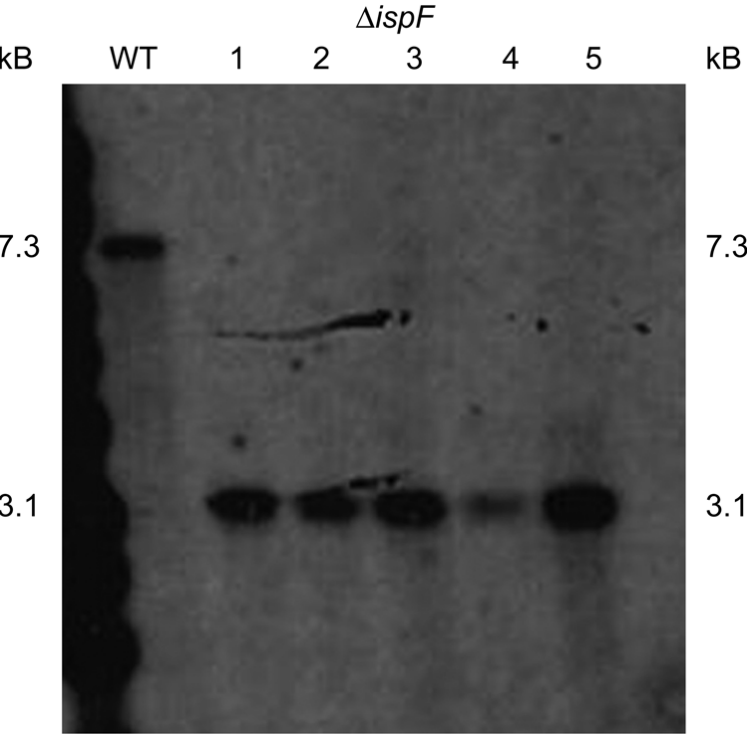
Southern blot of DispF complemented with pAPA3-*ispDF *(lanes 2–5) and WT (lane 1)- DNA was digested with *Bam*HI and probed with an upstream region of *ispF*. Expected size of WT band was 7.3 kb; DispF mutants were shown to only to possess the deletion band (3.1 kb), thus confirming the deletion of *ispF*.

We exploited our previously described methods [25-29] to determine whether *ispF *was essential in *M. tuberculosis*. Initially, we attempted to construct a knockout mutant using a two-step homologous recombination procedure. The two-step method employed the use of a suicide (non-replicating) construct containing an in-frame deletion of the *ispF *gene (Figures 2, 3). The construct (p2NIL-Δ *ispF*) was introduced into wild-type *M. tuberculosis *and single crossover (SCO) recombinant strains obtained. One SCO strain was used to isolate double crossover (DCO) recombinants; in the absence of antibiotic selection, DCO recombinants could have either the wild type or the deletion alleles. We screened 24 DCO recombinants; all had the wild-type gene.

The failure to isolate a deletion strain suggested that *ispF *is essential in axenic culture. To verify this hypothesis, we made a merodiploid strain in which an additional functional copy of *ispD *and *ispF *were introduced into the SCO strain on an L5-derived integrating vector under the control of the mycobacterial antigen 85A promoter (pAPA3-*ispDF*). The resulting strain had one deleted and two functional copies of *ispF*. Double crossovers generated from the merodiploid strain were isolated as before. Screening by PCR demonstrated that 19/24 DCOs had the wild-type gene and 5/24 had the deletion allele (*p *= 0.04, Fisher's exact t-test). The genotypes of the transformants with the deletion allele were confirmed by Southern hybridization (Figure 3). Since the chromosomal copy of *ispF *could only be deleted when a second functional copy was provided, this proved the essentiality of *ispF *in *M. tuberculosis*.

### A *Mycobacteria *model for structure-based studies

**Figure 4:**
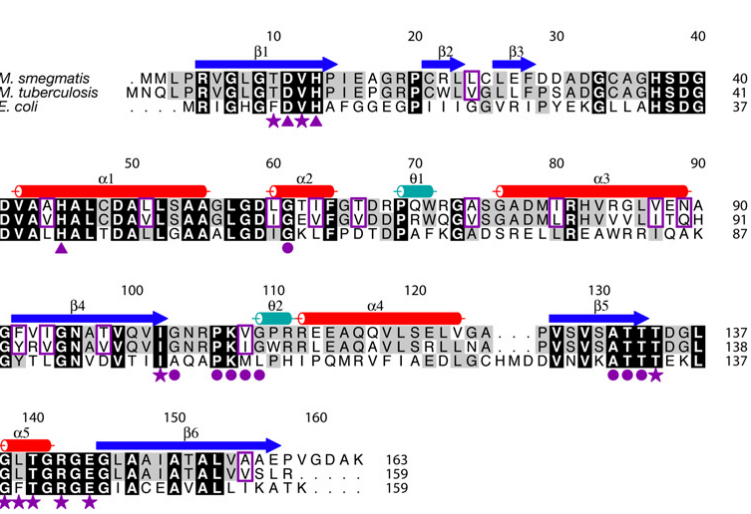
IspF homology. Amino acid sequence alignment of *Ms*IspF, *Mt*IspF, and *Ec*IspF. Secondary structure elements of *Ms*IspF are shown above the sequence. β-strands are blue, α-helices red, and 3_10_-helical segments aquamarine. *Ms*IspF and *Ec*IspF are aligned based on a structural overlay. Residues boxed in black are strictly conserved and those in grey are identical in two of the three sequences; similar residues from the *Mycobacteria *sequences are outlined in a purple box. ▲ identifies residues that interact with Zn^2+^, τ with Mg^2+^, H with CDP, and ♥ with the 2*C*-methyl-D-erythritol moiety of substrate; λ denotes residues that line the hydrophobic cavity. The residues that bind Mg^2+ ^and the ME2P fragment of substrate are based on observations in *Ec*IspF.

**Figure 5:**
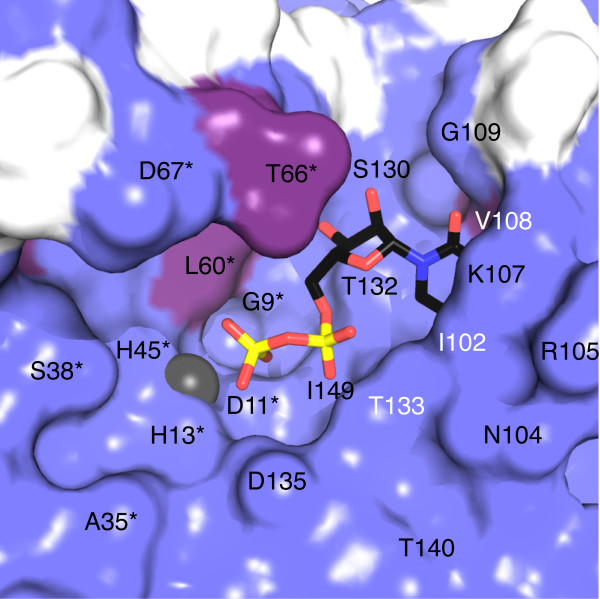
The van der Waals surface of the active site colored according to shared sequence identity with *Mt*IspF. Identical residues are colored slate-blue and similar residues are purple. The active site Zn^2+ ^is a grey sphere, and CDP is shown as a stick model with C atoms in black, N blue, O red, and P yellow. An asterisk indicates contributions from an adjacent subunit.

**Figure 6:**
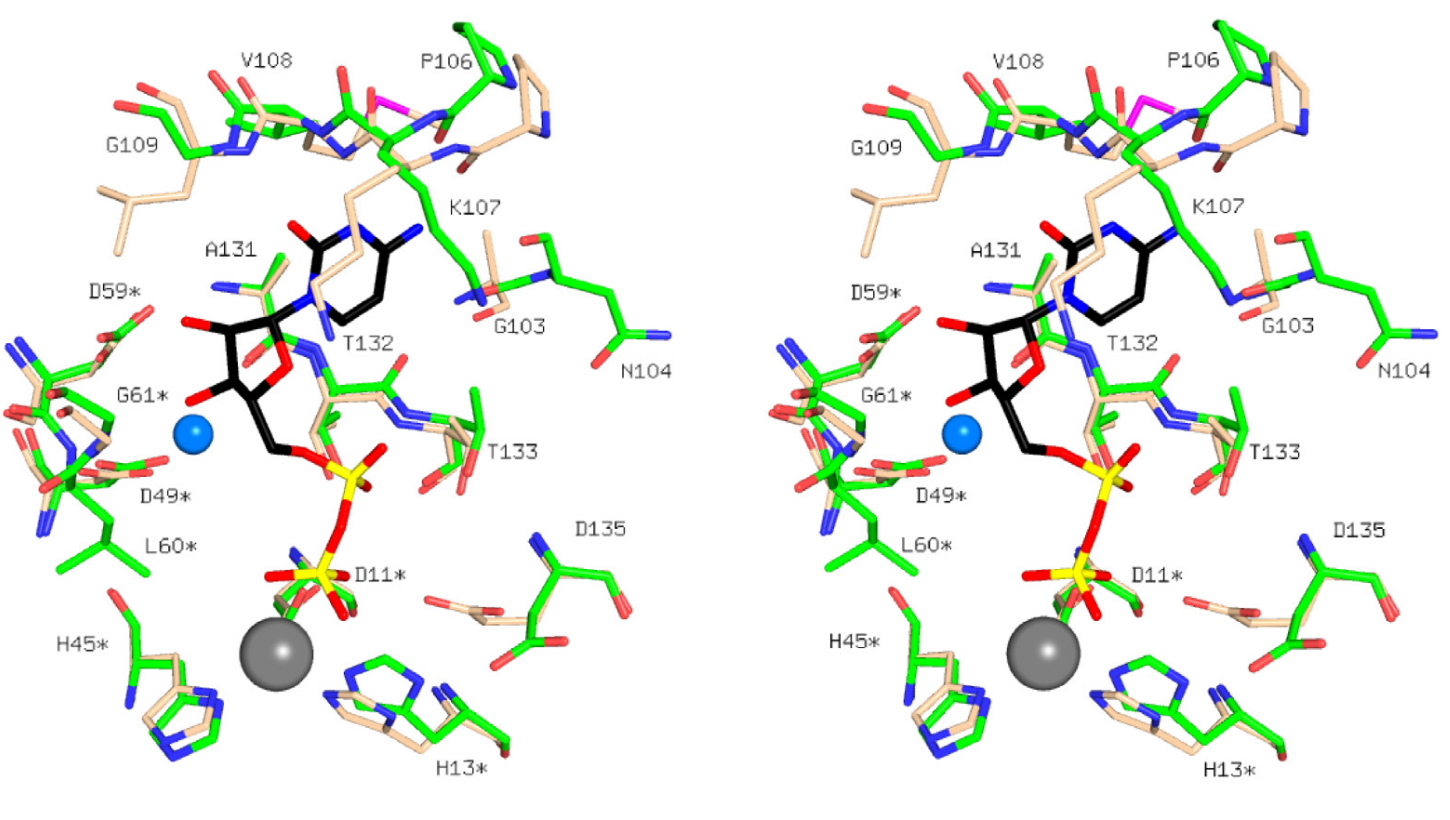
Stereo-view of the active site overlay of *Ms*IspF and *Ec*IspF. Residues from *Ms*IspF are labeled and CDP-B is the depicted conformer. Protein atoms are colored: C of *Ms*IspF green, C of *Ec*IspF wheat, all N atoms blue, O atoms red, and Se atoms magenta. The marine sphere depicts the water molecule that contributes to solvent-mediated interactions between IspF and the ribose hydroxyls of CDP.

That *ispF *is essential in *M. tuberculosis *validates the encoded enzyme as a chemotherapeutic target. We tried to determine the structure of *Mt*IspF to aid in rational ligand design, but the protein, though efficiently produced in recombinant form, was recalcitrant to crystallization. *Mt*IspF has 73% amino acid identity to *Ms*IspF, so we chose to study the orthologue on the basis that it would provide a suitable model of the pathogen enzyme. The recombinant *Ms*IspF is produced in high yield (approximately 30 mg L^-1 ^of bacterial culture), can be purified readily and provided well-ordered single crystals. A surface model of *Ms*IspF, which is colored by shared identity with *Mt*IspF, highlights the strong resemblance between these sequences, particularly at the active site (Figures 4, 5, 6). Generally, an accurate homology model is attainable in high sequence identity (>60%) cases [30,31] and the use of such models has been successful in structure-based ligand design. In certain cases, even with <60% sequence identity, homology models have been found useful. Examples being human carbonic anhydrase [32] and Rho kinase [33] where models were constructed from sequences that shared only 38% and 37% identity, respectively.

### Overall structure

The structure of *Ms*IspF bound to CDP was determined to a resolution of 2.2 Å. There are three subunits (chains A, B, and C) in the asymmetric unit, forming a homotrimer about a non-crystallographic axis. The model comprises residues 3–157 for each subunit, with residues 36–37 absent in chains A and B. Structures of several ligand-bound and native forms of IspF from *Campylobacter jejuni*, *E. coli*,*H. influenzae*, *Shewanella oneidensis*, and *Thermus thermophilus *are available in the Protein Data Bank [PDB, [15,34-37]]. *Ec*IspF [PDB code 1GX1, [16]] was chosen as the model for the structural comparisons to follow because it was built using high-resolution data (1.8 Å) and contains the ligand CDP. The two sequences share 38% identity, and the r.m.s.d. values for the superposition of the *Ms*IspF onto the *Ec*IspF trimer range from 1.10–1.16 Å, depending upon which chains are aligned.

**Figure 7:**
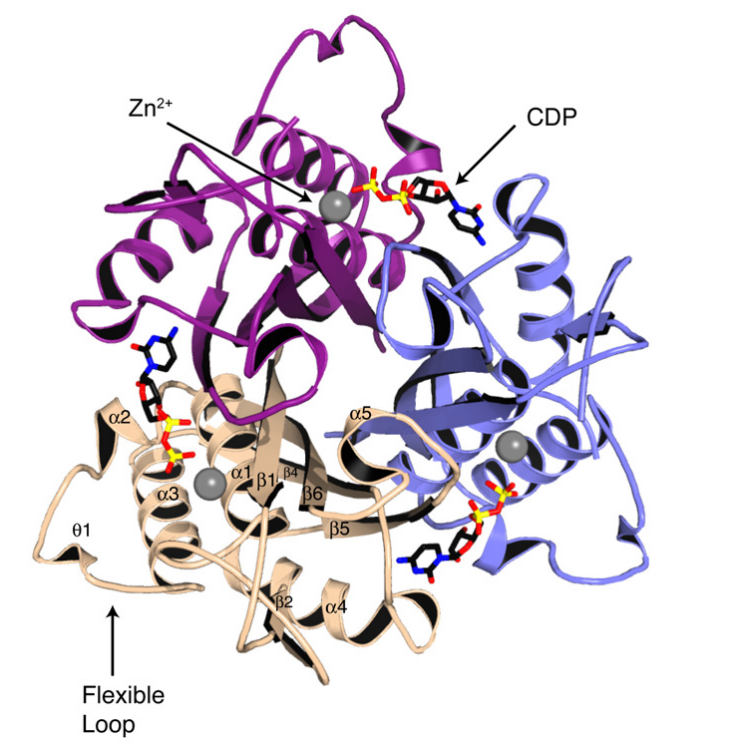
Ribbon diagram of the trimer. The *Ms*IspF trimer viewed down the molecular three-fold axis. The individual subunits are shown in slate, wheat, and purple. Selected secondary structure elements of the wheat subunit, CDP and Zn^2+ ^are depicted as in Figure 5.

**Figure 8:**
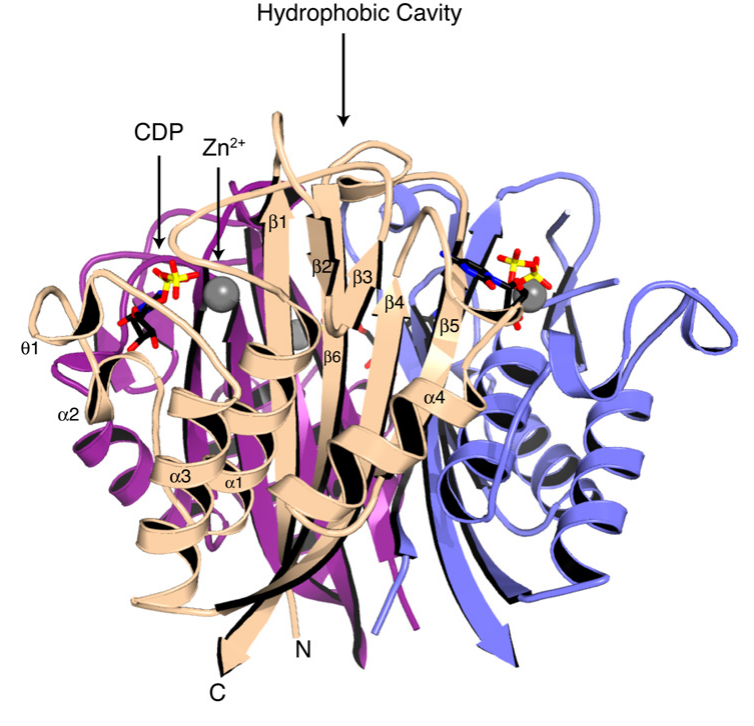
Ribbon diagram of the trimer. Orthogonal view compared to Figure 7.

*Ms*IspF closely resembles *Ec*IspF [16-18]. Each subunit displays an α/β fold which contains six β-strands, five α-helices, and two 3_10 _helices (Figures 4, 7, 8). Four of the strands (β1, β4–6) comprise a central β-sheet that packs against the α- and 3_10 _helices. The other two strands form a short sheet at the end of a loop that extends into the space between α1, α3, and α4. One 3_10 _helix (θ2) of *Ms*IspF overlays with that of *Ec*IspF (designated θ1 by [16]), but the second (θ1) occurs between α2 and α3 rather than following α4.

Trimer formation arises from edge-to-face packing of the β-sheets, with the largest section of the interface occurring between β1 and β5 of adjacent subunits (Figures 7, 8). Thus, the interior shape of the trimer resembles a trigonal prism whose faces are comprised of β-sheets from the individual subunits. The *Ms*IspF trimer has the same overall dimensions as *Ec*IspF, measuring approximately 40 Å in height along the three-fold symmetry axis and 60 Å in diameter at the widest point perpendicular to this axis. In addition, like the *E. coli *enzyme, most of the hydrogen bonds between the subunits involve side chain interactions. The trimer interface interactions also resemble those of *E. coli *in that they are primarily hydrophobic; approximately 65% of atoms comprising both of these enzyme interfaces are non-polar. *E. coli *and *M. smegmatis *are mesophiles. In contrast, only 58% of atoms in the interface of IspF from the thermophile *T. thermophilus *are non-polar [35].

### Hydrophobic cavity

At the center of the trimer is a hydrophobic cavity that opens toward the C-terminal ends of β1, β4 and β5. Side chains of residues Thr10, Val12, Ile102, Thr134, Leu139 and Thr140 from each subunit line the interior of the cavity while two arginines (Arg142 from subunits A and B) and the main chain of Gly138 and Leu139 of subunit C shape the aperture (data not shown). Arg142 is held in place through an electrostatic attraction to Glu144. In *Ec*IspF, a salt-bridge between Arg142-Glu144 from all three subunits forms the aperture. Here, subunit C is less ordered and this contributes to the observed asymmetry. The density is poorly defined between residues 137–144 in subunit C and the average thermal parameter for this region (58.4 Å^2^) is much higher than in subunits A (34.6 Å^2^) and B (18.1 Å^2^).

The distance from the base of the cavity to the opening (16 Å) and the diameter of the aperture (6 Å) are comparable to those observed in *Ec*IspF. The volume of the cavity of *Ms*IspF (1940 Å^3^), however, is significantly larger than that of *Ec*IspF (1540 Å^3^). In *Ec*IspF the cavity is ellipsoidal and the floor parabolic; the major axis of the ellipsoid runs from the aperture to the floor of the cavity [15]. In *Ms*IspF the cavity is trigonal pyramidal, with the aperture corresponding to the tip of the pyramid and the floor to the base. Residue differences in the lining of the cavity contribute to shape and diameter variation. In *E. coli*, the cavity is lined with the side chains of six large hydrophobic residues, Phe7 and Phe139 from each subunit, whereas the corresponding residues in *Ms*IspF are Thr10 and Leu139. In *Ec*IspF, the floor of the cavity is sealed by three His5-Glu149 salt bridges [15]. Hydrophobic interactions seal the floor of *Ms*IspF. Here, residues Leu8 and Ile149 replace the *Ec*IspF salt-bridge. The cavity in *Mt*IpsF should bear a strong resemblance to that in *Ms*IspF since the residues that contribute to the lining (discussed above) are strictly identical in the two sequences (Figure 4).

In common with crystal structures of other IspF trimers, non-protein electron density was observed in the hydrophobic cavity of *Ms*IspF. In *Ec*IspF, phosphate, farnesyl pyrophosphate, GPP, and IPP have been shown to bind within this cavity [15]. There is as yet no evidence to prove that ligand binding here regulates enzyme activity. The cavity is distant from the three catalytic sites but since, as will be explained, oligomerisation is required to generate the functional enzyme then occupancy of the hydrophobic cleft may contribute to the stability of the IspF trimer.

In *Ms*IspF the density observed in the cavity is diffuse and we presume that a similar mixture of ligands may be present. IPP was modeled into this density at 50% occupancy based on fit and ligand identification in the *Ec*IspF cavity. Although a methodical and thorough approach was used in fitting the ligand, the thermal parameters of IPP (47.5 Å^2^) exceed the average of the protein (27.5 Å^2^). The ligand-protein interactions, though not clearly defined, do resemble those observed in *Ec*IspF [PDB code 1H47, [15]]. The guanidino groups of Arg142 from two subunits bind to the β-phosphate; in *Ec*IspF, the side chain from the corresponding residue (Arg142) of all three subunits contributes to this interaction. In *Ms*IspF, the bridging phosphodiester oxygen of IPP binds to the amide of Leu139 in subunit C and one of the α-phosphate oxygens binds to the main chain amide of Leu139 in subunit B. In *Ec*IspF, these ligand atoms interact with the main chain amide of the corresponding residue (Phe139) of all three subunits.

### Active site

There are three active sites in the trimer, each located at the interface between two adjacent subunits. The active site (Figures 5, 6) comprises a rigid nucleotide and cation (Zn^2+ ^and Mg^2+^) binding pocket and a flexible loop for binding the ME2P moiety of substrate [16-18,36]. Only one of the two cation-binding sites, the Zn^2+ ^site, is occupied here [8,15,16]. This cation is approximately 75% occupied in two subunits and 50% occupied in the third. The Zn^2+ ^displays tetrahedral coordination, in similar fashion to that observed in other IspF structures, by Asp11, His13, His45, and the β-phosphate of CDP. In the higher resolution model of *Ec*IspF, the second cation (Mg^2+ ^or Mn^2+^[16,18]) is coordinated by the side chain of Glu135 and two oxygens from the diphosphate of CDP. In *Ms*IspF and *Mt*IspF, the glutamate is replaced with aspartate. This residue is strictly conserved as an aspartate or glutamate across 450 IspF sequences (data not shown), suggesting that a negative charge is required to coordinate the second cation and that either negatively charged amino acid will suffice. In the structure of *Ms*IspF, the lower resolution data or CDP disorder may preclude identification of the second cation (see below).

Two conformers of CDP, each at approximately half occupancy, are present in each of the three active sites of the trimer. We only show one conformer in Figures 5 and 6 for the purpose of clarity. In the conformers, the ligand-protein interactions are maintained for the pyrimidine and the ribose but diverge at the diphosphate. The average thermal parameters of the conformers, hereby referred to as CDP-A and CDP-B, are 20.8 and 29.2 Å^2^, respectively. The presence of CDP disorder is likely linked to the incompletely occupied Zn^2+ ^binding site. The mode of ligand binding of CDP-B more closely resembles that observed in *Ec*IspF (Figure 6). In this mode, three interactions are present between the protein and ligand diphosphate. Two of these are hydrogen bonds formed between the α-phosphate and the side chain hydroxyl and main chain amide of the strictly conserved Thr133. The third is ligand-metal ion coordination between the β-phosphate and the active site Zn^2+^. The interaction between the Zn^2+ ^and the β-phosphate is preserved in CDP-A, but an additional hydrogen bond occurs between the β-phosphate and the hydroxyl group of Thr132. In CDP-A, the α-phosphate also forms a hydrogen bond with the side chain hydroxyl of Thr133, but the bridging diphosphate oxygen interacts with the main chain amide of this residue and the side chain of Thr132 instead.

The architecture of the active site at the cytosine and Zn^2+ ^binding sites and the interactions formed with CDP by *Ms*IspF are similar to that observed in *Ec*IspF. Furthermore, *Ms*IspF residues that contribute to this binding site are all identical or conserved in *Mt*IspF (Figure 4). The cytosine is bound in an aliphatic pocket created by side chains of residues from β5 and the loop between β4 and θ2 from a single subunit. The cytidine is stacked between the side chains of Ala131 and Lys107, which are strictly conserved in *E. coli *and *M. tuberculosis*. Both binding sites in *Ec*IspF and *Ms*IspF are characterized by four hydrogen bonds between the pyrimidine and main chain atoms of the protein. In *Ms*IspF, these backbone atoms are from residues Gly103, Pro106, Val108 and Gly109, and, in *Ec*IspF, Ala100, Pro103, Met105, and Leu106. These residues are strictly conserved in *Ms*IspF and *Mt*IspF with the exception that *Mt*IspF Ile109 replaces *Ms*IspF Val108 (Figure 4).

Because the interactions involve backbone atoms, high conservation of these residues is not necessarily required. The critical elements required to maintain similar protein-ligand interactions are the shape and size of the cytosine pocket. Two pairs of hydrophobic interactions contribute to this function in *Ms*IspF. One pair of hydrophobic interactions occurs between the side chains of Pro106 and Leu146 and the second between the side chains of Val101 and Val108. Both sets of residues are highly conserved (>85%) in 450 IspF sequences, including both *E. coli *and *M. tuberculosis*. The first is conserved as a proline-leucine/isoleucine pair and the second as two aliphatic residues, where the identities of the residues are leucine, methionine, valine, isoleucine, or phenylalanine.

The ribose hydroxyls are oriented by several hydrophilic interactions involving strictly conserved residues in *Ms*IspF, *Ec*IspF and *Mt*IspF. The ribose hydroxyls form hydrogen bonds with the side chain of Asp59* (the asterisk denotes contributions from another subunit) and the amide of Gly61*, and solvent-mediated interactions are observed with the side chain of Asp49* and the carbonyl of Ala131 (residues Asp56*, Gly58*, Asp46* and Ala131 in *Ec*IspF, respectively). Moreover, in *Ms*IspF and *Ec*IspF, the side chain orientation of Asp59* is maintained through hydrophilic interactions. Here, this aspartate accepts hydrogen bonds donated by amides of Gly61*, Thr62*, and Ala131 and the side chain of Thr62*, and, in *Ec*IspF, with the amides of Gly58*, Lys59*, and Ala131. The *Ms*IspF residues that contribute to the orientation of Asp59* are strictly conserved in the sequence of *Mt*IspF except for Thr62*, which is a glutamate in the latter. Main chain atoms are the primary contributors to stabilization of Asp59*, so this amino acid replacement is unlikely to affect conformation or function.

The nucleotide-binding pocket is only part of the active site. In *Ec*IspF the remaining fragment of substrate, ME2P, is bound by contributions from α2, α3, and residues 33–37, and a flexible loop, which comprises residues 61–71 [16-18]. The largest Cα r.m.s.d. differences between *Ec*IspF and *Ms*IspF occur in this loop. In *Ec*IspF, the loop is stabilized by hydrogen bonds between the side chain of His34* and the carbonyl atoms of Asp63* and Asp65*. His34* is conserved in *Ms*IspF (His37*), but the aspartates are not. Here, no well-defined density is observed for His37* in two of the trimer subunits. In the third, the side chain of this residue forms hydrogen bonds to the main chain carbonyl of Arg68* and the side chain of Asp67*. The former resembles the *Ec*IspF His34*-Asp65* interaction, but the latter reflects the different conformations of this loop present in the two orthologues. This loop is further stabilized in *Ms*IspF by a hydrophilic interaction between the carbonyl of Ile63* and the side chain of Arg68*, a residue which is not conserved in *Ec*IspF. The stabilization of the loop through hydrogen bonding to the side chain of an aspartate as observed in *Ms*IspF can be maintained in *Mt*IspF as this residue is identical, but the arginine is replaced by a second aspartate. Although the main chain interactions might be preserved by an aspartate, the side chain interactions could not.

ME2P is oriented by several hydrophobic and hydrophilic interactions with *Ec*IspF [18]. The amides of Ser35* and His34* and the hydroxyl of Ser35* form hydrogen bonds with oxygens of the attacking 2-phosphate group. The identities of these residues and the positions of the residues that bind and orient the attacking 2-phosphate group are maintained in *Ms*IspF (Figure 4). In *Ec*IspF, the side chains of Ile57* and Leu76* make van der Waals contacts with the methyl group of ME2P. These residues are replaced by another hydrophobic pair, Leu60* and Met78*, in *Ms*IspF. When a model of *Ec*IspF containing substrate [PDB code 1U43, [18]] is superimposed onto *Ms*IspF, these residues are able to maintain contact with the methyl group of ME2P. The 3-hydroxyl group of the ligand interacts with the carbonyl of Phe61 in *Ec*IspF. This residue is part of the flexible loop, and the equivalent residue in *Ms*IspF (Phe64*) does not maintain this interaction in the superposition. Phe64* is preceded by a glycine in *Ms*IspF and a proline in *Ec*IspF. Glycine flexibility would permit a conformational change to accommodate interactions between Phe64* and the ligand. Alternatively, there is a hydrogen bond present between the carbonyl of Gly65* and the 3-hydroxyl group of the superimposed substrate. The aforementioned residues corresponding to those observed in *Ms*IspF are all identical in *Mt*IspF except for Leu60*, which is an isoleucine instead. The binding component of this residue, the side chain hydrophobicity, is maintained in *Mt*IspF, as this residue is also an isoleucine in *E. coli *and is strictly conserved as isoleucine, leucine, or valine in 450 IspF sequences.

## Conclusion

There is an urgent need to identify new targets and to develop new treatments for tuberculosis. Our work demonstrates that *ispF *is essential in *M. tuberculosis*, thus establishing it as a potentially valuable target for chemotherapeutic intervention. In addition, we have determined the crystal structure of the closely related orthologue *Ms*IspF bound to CDP. The protein is a homotrimer with three equivalent active sites formed at the subunit interfaces. Each active site bears a strong resemblance to those observed in other IspF structures, presenting a rigid CDP-Zn^2+ ^binding-pocket and a flexible substrate-binding loop. *Mt*IspF and *Ms*IspF share 73% sequence identity, and, of the eleven residues in the active site that bind CDP, ten are identical and the eleventh highly conserved. Based on the high degree of similarity between the orthologues, particularly in the active site, the structure of *Ms*IspF provides a suitable template for structure-based inhibitor design targeting the pathogenic organism *M. tuberculosis*.

## Methods

### Culture and manipulation of *M. tuberculosis *and *M. smegmatis*

*M. tuberculosis *(H37Rv) was grown on Middlebrook 7H10 agar or Middlebrook 7H9 broth (with 0.05% Tween 80), with 10% OADC (oleic acid, bovine serum albumin, dextrose, catalase) supplement (Becton Dickinson). *M. smegmatis *(ATCC 700084) was grown on Lemco medium (5 g l^-1 ^Lemco powder, 5 g l^-1 ^NaCl, 10 g l^-1 ^Bacto peptone) with 0.05% Tween 80 (liquid) or 15 g l^-1 ^agar (solid).

### Plasmids for *M. tuberculosis ispF *knockouts

The deletion delivery vector was constructed as follows: PCR was used to amplify the regions either side of *ispF *using the primer pairs IspFNFor/IspFNRev and IspFCFor/IspFCRev (Table 1) and the resulting products were subcloned with the Zero Blunt^® ^TOPO^® ^PCR Cloning Kit (Invitrogen). The DNA fragments were gel purified (Qiagen Qiaquick Gel Extraction Kit) and then cloned into p2NIL [29] to generate a deletion of *ispF *in which 379 bp of the gene was absent. The marker gene cassette from pGOAL19 [29] was then cloned into the unique *Pac*I site to generate the final delivery vector, p2NIL-Δ *ispF*.

**Table 1:** Primers used in this study

**Primer**	5' Sequence*
IspFNFor	AAGCTTgtacgagttcccgctgaaaacgc
IspFNRev	GGATCCgagagtctgcccgtcgagctg
IspFCFor	GGATCCgcaatcgctacggcattggtggt
IspFCRev	GGTACCaccaccaccgacgggctgggc)
IspDFSh	TTAATTAAgacgccaaagccgagaccatcctt
IspDFRev	TTAATTAAgccagcttacctgcccaattgctg
IspFIntA	ggtcgaatcgcactgacac
IspFIntB	cgatcatctgggtgatatgc
MsIspFNter	CATATGatgttgcctcgcgtagggc
MsIspFCter	GGATCCtactttgcatcaccgaccggtt
IspDUSFor	gacgagaatcaatgagacct
IspDUSRev	agtgatatcggctcggtgac

*Upper case letters indicate inserted restriction sites.

To make the complement vector (pAPA3-*ispDF*) used to generate the merodiploid strain, part of the operon, which includes both the *ispD *and *ispF *genes, was amplified by PCR using the primer pair IspDFSh/IspDFRev (Table 1) and subcloned as *Pac*I fragments into the integrating vector pAPA3 [28]. The integrity and directionality of all constructs were confirmed by DNA sequencing.

### Isolation and genotyping of recombinant strains

A single crossover strain was generated by electroporating *M. tuberculosis *with 1 μg plasmid DNA and recombinants selected on 100 μg/ml hygromycin, 20 μg/ml kanamycin and 50 μg/ml X-gal as previously described [26]. A single strain was streaked out in the absence of any antibiotics to allow the second crossover to occur. Double crossovers were selected and screened for using 2% w/v sucrose and 50 μg/ml X-gal; white colonies were patch tested for kanamycin and hygromycin sensitivity to ensure that they had lost the plasmid during homologous recombination. PCR was used to determine the presence of the wild type or deletion allele using primers IspFintA and IspFintB (Table 1), which amplify 1.4 kbp and 1 kbp fragments from the wild type and deletion alleles respectively.

To generate the merodiploid strain, the pAPA3-*ispDF *plasmid was electroporated into the single crossover strain and recombinants isolated on 10 μg/ml gentamicin, 100 μg/ml hygromycin, 20 μg/ml kanamycin and 50 μg/ml X-gal. A single recombinant was streaked out without antibiotics to allow a second crossover to occur, and double crossovers were isolated as before, except that gentamicin was included at all stages. PCR and Southern blot analysis were used to confirm the double crossover deletion allele (delinquent mutant) generated from the merodiploid strain.

### Southern analysis

To generate a probe for Southern analysis, the region upstream of the *ispD *was PCR-amplified using primers IspDUSFor and IspDUSRev (Table 1) and the isolated fragment labeled with AlkPhos Direct system (GE Healthcare). Genomic *M. tuberculosis *DNA (2 μg) was digested with *Bam*HI; the digestion products were separated on an agarose gel and transferred by vacuum blotter onto a Hybond N+ membrane (GE Healthcare). The membrane was hybridized for 16 h in Alk Phos Direct hybridization buffer (GE Healthcare) at 65°C with the labeled probe. Primary and secondary post hybridization washes were carried out (two primary washes for 10 min each at 55°C and two secondary for 5 min each at RT, as per manufacturers' instructions), and the probe detected by CDP-Star (GE Healthcare).

### Cloning and expression of *M. smegmatis ispF*

The *ispF *gene was amplified by PCR from genomic DNA, previously obtained with an established protocol [38], using the primers *Ms*IspFNter and *Ms*IspFCter (Table 1) and cloned into *Nde*I/*Bam*HI-digested pET15b_TEV, a modified pET15b (Novagen) expression vector that includes an N-terminal tobacco etch virus (TEV) protease cleavage site in place of the thrombin cleavage site. The integrity of the pET15b_TEV-*ispF *construct was confirmed by sequencing.

This construct was chemically transformed into BL21(DE3) Gold cells (Stratagene) and selected for on Luria-Bertani (LB) agar plates containing carbenicillin (50 μg/ml). A single colony was cultured at 37°C to an *A*_600 _of ~0.6 in 1 L of LB containing carbenicillin (50 μg/ml) and transferred to an ice water bath for 20 minutes. Subsequently, 1 mM isopropyl β-D-1-thiogalactopyranoside was added to induce expression and the culture was incubated at 22°C overnight. Cells were harvested by centrifugation and stored at -20°C.

### Purification of *Ms*IspF

The cell pellet was resuspended in 30 mL of binding buffer (500 mM NaCl, 20 mM Tris-HCl, pH 8, 15 mM imidazole) containing lysozyme and DNAse I [16] and lysed using a One-shot cell disruptor (Constant Cell Disruption Systems). The soluble fraction was isolated by centrifugation (48,400 *g*, 30 minutes at 4°C), passed through a 0.2 μ filter, and loaded onto a 5 mL HisTrap HP column (GE Healthcare) loaded with Ni^2+ ^and equilibrated in binding buffer. The protein was eluted using a combination of step and linear gradients from 0 to 500 mM imidazole and concentrated to 873 μM (theoretical ε_0 _= 6990 M^-1 ^cm^-1 ^including the His-tag). The His-tag was cleaved with TEV protease (2 mg at 22°C for 12 hours). The sample was subsequently dialyzed into 50 mM NaCl, 20 mM Tris-HCl, pH 8 and 1 mM dithiothreitol, passed over a 5 mL HisTrap HP column to remove TEV protease and uncleaved protein, and further purified by anion exchange chromatography (5 mL Q HP, GE Healthcare). The sample was then dialyzed into 50 mM NaCl, 10 mM Tris-HCl, pH 8, 2 mM MgCl_2 _and concentrated to 405 μM (theoretical ε_0 _of 5500 M^-1 ^cm^-1 ^excluding the His-tag). This protein solution was used for crystallization. The high degree of sample purity was confirmed by SDS-PAGE and matrix-assisted laser desorption ionization-time-of-flight mass spectrometry.

### Crystallization and data collection

Prior to crystallization, the protein was incubated with 5 mM CDP at 4°C for 12 hours. Crystals were grown in three days by sitting drop vapor diffusion at 20°C using 0.8 μL of protein solution and 0.8 μL of reservoir (18% PEG 8000, 0.1 M sodium cacodylate pH 6.5, 0.2 M calcium acetate). A single crystal (50 × 50 × 50 μm) was cryo-protected in reservoir adjusted to include 18% glycerol and flash-cooled at -173°C. Diffraction data were collected (Table 2) at the European Synchrotron Radiation Facility (ESRF), station ID 23-2, on a MarMosaic 225 CCD detector at a wavelength of 0.8730 Å. The data were integrated, merged, and scaled using MOSFLM [39] and SCALA [40] from the CCP4 suite of programs [41].

**Table 2:** Crystallographic statistics

Structure	*M. smegmatis *IspF
Unit cell, *a, b, c *(Å)	159.1, 159.1, 54.2
Resolution range (Å)	51.3-2.2 Å
No. of observations	117,125
No. of unique reflections	33,193
Completeness (%)	95.4 (76.3)^†^
<*I*/*σ(I)*>	6.0(1.7)
R_sym _(%)	8.1(34.6)
Multiplicity	3.5(2.1)
Wilson *B *(Å^2^)/DPI (Å)^‡^	28.1/0.16
Protein residues	461
Water molecules	272
CDP/Zn^2+ ^molecules (occupancy)	3(1)/3(0.75/0.5/0.75)
IPP molecules (occupancy)	1(0.5)
PEG/Glycerol/Ethylene glycol/Acetate molecules	2/3/2/1
R_work_/R_free _(%)	16.3/20.6
Average *B *(Å^2^)	
Subunit A/B/C overall	30.7/19.7/31.8
All main/side chain	25.9/29.3
Waters	35.8
CDP/Zn^2+^	25.0/36.0
IPP	47.5
PEG/Glycerol/Ethylene glycol/Acetate	45.3/58.4/28.5/38.9
RMS bond lengths (Å)	0.013
RMS bond angles (°)	1.570
PDB Code	2UZH

^† ^Values in parentheses pertain to the highest resolution shell (width = 0.11 Å). *B *is the isotropic thermal parameter.

^‡ ^DPI = diffraction-component precision index [53].

### Structure determination and refinement

The crystal belongs to space group *I*4 and has three subunits in the asymmetric unit. The structure was solved by molecular replacement with AMORE [42] using an *Ec*IspF trimer as the search model [PDB code 1GX1, [16]]. *Ec*IspF shares 38% amino acid identity with *Ms*IspF. Search model bias was removed/reduced with prime-and-switch phasing and a partial *Ms*IspF model was built using RESOLVE [43]. Restrained maximum likelihood refinement was done using REFMAC5 [44] and PRODRG [45] to provide ligand dictionaries. Non-crystallographic symmetry restraints were imposed early on but removed at later stages of refinement. The R_free _calculation was performed on 5% of the data. COOT [46] was used to inspect Fourier syntheses and manipulate the model during refinement. The occupancies for CDP and Zn^2+ ^were based on consideration of refined thermal parameters and the appearance of electron and difference density maps. Statistics for the model are presented in Table 2.

### Model analysis

Root-mean-square deviation (r.m.s.d.) values for superpositions were calculated using LSQMAN [47]. The values for the superpositions of chain A onto B, A onto C, and B onto C, respectively, were 0.54 Å, 0.59 Å, and 0.67 Å over 153 Cα atoms. Analysis of model geometry with PROCHECK [48] demonstrated that all residues are within allowed regions of the Ramachandran plot. Secondary structure assignments were made using DSSP [49], COOT, and by visual inspection. The trimer interface was analyzed using the Protein-Protein Interaction Server [50]. The volumes of the hydrophobic cavities at the trimer centers were calculated and refined with VOIDOO [51] using a rolling probe with a radius of 1.4 Å. Figure 1 was created with ChemDraw, Figure 2 with Adobe Illustrator and Photoshop, 4 with Aline (C. S. Bond and A. W. Schüttelkopf, personal communication), and 5–8 with PyMOL [52].

## Authors' contributions

LB carried out cloning, biochemical and crystallographic experiments, ACB carried out all research on *M. tuberculosis*. TP planned and supervised all work with *M. tuberculosis*. WNH conceived of the study, and together all authors participated in its design and coordination. All authors contributed to data interpretation and in writing the manuscript.
